# Oral Cancer Awareness Among Princess Nourah bint Abdulrahman University Dental Students and Interns

**DOI:** 10.7759/cureus.46280

**Published:** 2023-09-30

**Authors:** Farida Fahad Bsher, Hend Waguih Salem, Sahar ElRefai

**Affiliations:** 1 Department of Basic Dental Sciences, College of Dentistry, Princess Nourah bint Abdulrahman University, Riyadh, SAU

**Keywords:** knowledge, awareness, intern, dental student, oral cancer

## Abstract

Background: Dentists have a very important role in the early diagnosis of oral cancer premalignancy. This aspect of dental practice requires an adequate level of knowledge.

Objective: This study aimed to assess the level of knowledge of undergraduate dental students and dental interns about early diagnosis and risk factors of oral cancer.

Methods: The present cross-sectional study was conducted at the Dental College, Princess Nourah bint Abdulrahman University, Saudi Arabia. Fourth-year and fifth-year students and dental interns were enrolled in the study. The participants were recruited using a convenient, non-probability sampling method. A total of 103 participants filled out a close-ended, online questionnaire. We used descriptive and analytical statistics to analyze the responses to the questionnaire.

Results: The level of general knowledge was moderate (67%). More than half of the participants gave complete responses to questions related to early signs of oral cancer (67%), risk factors (54%), risk sites (75%), treatment plans for leukoplakia (66%), and unhealed ulcer management (58%). We found significant associations between the correct responses and the year of the study regarding early signs of oral cancer (p=0.0001), high malignant potential lesions (p=0.00001), and chronic unhealed ulcer management (p=0.015).

Conclusion: A more satisfactory level of awareness is needed among future dentists, to prevent missing patients undiagnosed with early oral cancerous lesions during routine screening. Consequently, upgrading theoretical curricula, clinical experience, and post-graduate participation in cancer prevention programs is highly recommended.

## Introduction

Globally, oral cancer is ranked as the tenth most common cancer, and its incidence is growing in most countries, particularly in developing countries [1]. Oral cancer is a major health problem in Asia, with the highest frequency recorded in the South-East Asia region (6.4 per 100,000). In the Kingdom of Saudi Arabia, oral cancer is a very common malignancy (17.6%), accounting for as much as 26% of head and neck cancers [2], and its prevalence ranges from 21.6% to 68.6% [3].

Epidemiological studies have shown that smoking and alcohol intake are the primary risk factors, and they play a great role in its high incidence, particularly among younger individuals. Smokeless tobacco use (Shamaa and Qaat chewing) also represents a significant risk factor for oral and pharyngeal cancer [4].

Parakh and coworkers [5] have shown that the greatest challenge is that oral cancer is not detected early enough for successful treatment. Although oral malignancy is mostly a visible lesion, there is a lack of awareness about the presentation of oral cancer, the potentially malignant lesions, and associated risk factors. The delayed diagnosis is associated with a high rate of morbidity and mortality.

Su et al. [6] have emphasized that appropriate and early diagnosis of the condition in early stages can improve patient outcomes; such improvement can be up to a five-year in survival rate. It has been observed that the five-year survival rate of oral cancer is only 50%, which can be improved to 80% if the lesion is diagnosed at an early stage. Furthermore, when patients are diagnosed on time, they have the best chance for cure with a lower treatment costs and improved quality of life.

Dental practitioners have a substantial role in diagnosing oral cancer. Previous reports have shown that a high number of general dental practitioners are not able to adequately detect oral cancer in its early stages due to a lack of knowledge and incompetent attitudes. Therefore, a more serious approach to the examination of the oral cavity with the aim of early detection of malignant changes in the oral mucosa is greatly needed [7-9].

This study aimed to assess the level of knowledge of undergraduate dental students and dental interns about the early signs and symptoms of oral cancer, the lesions with high malignant potential, risk factors, and the major factors that affect the prognosis of oral cancer.

## Materials and methods

Ethical considerations

This study obtained ethical approval from the Institutional Review Board of the College of Dentistry, Princess Nourah bint Abdulrahman University (PNU), Riyadh, Saudi Arabia. An informed consent was attached to the questionnaire. We informed the students and interns that their participation is voluntary. Subjects who decided to fill out the questionnaire should provide their agreement to participate in the study. We confirmed to the participants that we would use their anonymized data only for research purposes and we would maintain their confidentiality.

Study design

This was a cross-sectional, questionnaire-based survey study.

Study setting and date

The study was conducted at the College of Dentistry, Princess Nourah bint Abdulrahman University (PNU), Riyadh, Saudi Arabia between April 2020 and November 2020.

Eligibility criteria

The questionnaire was presented to all fourth- and fifth-year dental students as well as dental interns. Individuals who agreed to complete the questionnaire were included in the study. Students of the first, second, and third years, those who refused to participate, or gave incomplete responses were excluded. The rationale for choosing the fourth- and fifth-year undergraduate students is their clinical exposure with expected sufficient knowledge regarding oral cancer.

Study instrument

An online, self-administered questionnaire was administered to fourth- and fifth-year dental students and interns in the College of Dentistry, PNU, Riyadh, Saudi Arabia. Approximately 10 minutes were required for the completion of the questionnaire. Our survey was based on a pre-validated questionnaire that had been developed from an earlier study [10].

The first part of the questionnaire includes the demographic details of the participant, such as age and year of study. The second part of the questionnaire includes an inquiry about the frequency of the patient’s oral mucosa examination, followed by seven questions related to knowledge regarding the clinical appearance of early oral cancer, risky sites, most important risk factors, lesions having a high malignant potential, major factors affecting the prognosis of oral cancer, what would they do to a patient with unhealed ulcer persisting more than two weeks, and the treatment plan for leukoplakia.

Data collection

After obtaining ethical approval, the investigators collected the participants’ data using an online, self-administered questionnaire. It was generated using Google Forms, and the link was emailed to all the students, with the purpose of the survey explained to them. A convenient, non-probability sampling method was applied for recruiting the participants.

Statistical analysis

All data were tabulated and analyzed by the statistical package for the social sciences software program, IBM SPSS Statistics for Windows, version 22 (IBM Corp., Armonk, NY, USA). We presented categorical data as frequencies and percentages and analyzed the likely associations between the correct responses to the knowledge questions and the age groups, or the year of study of the participants using the Pearson’s Chi-square test. For evaluation of the knowledge level, every correct response to the knowledge questions received one point, while the incorrect response received zero. So, the total knowledge score ranged from 0 to 7. The cut-off of 4 points, representing approximately 60% (4/7), was defined. A score of less than 4 represented poor knowledge about oral cancer and a score of more than 4 represented good knowledge. A p-value <0.05 was considered statistically significant.

## Results

The total participant number was 103. Most (79%) of them were aged 23-25 years. They included fourth- and fifth-year students (39% and 28%, respectively) and dental interns (33%). Most (80%) of them always examine the patients’ oral mucosa (Table 1).

**Table 1:** Demographic Characteristics of the Study Participants and the Frequency of the Patient’s Oral Mucosa Examination (N=103)

Variables		N	%
Age group, year	20-22	20	19%
	23-25	81	79%
	Above 25	2	2%
Year level	Fourth-year dental student	40	39%
	Fifth-year dental student	29	28%
	Dental intern	34	33%
How often do you examine patient's oral mucosa?	Always	82	80%
	Often	15	15%
	Sometimes	6	6%
	Never	0	0%

The level of general knowledge was high with a percentage amounting to 67%. More than half gave correct responses to questions related to early signs of oral cancer (67%), risk factors (54%), risk sites (75%), treatment plans for leukoplakia (66%), and unhealed ulcer management (58%). We observed a high frequency (96%) of correct responses to the major factor affecting the prognosis of oral cancer; however, less than half (41%) correctly identified the lesions with high malignant potential (Table 2).

**Table 2:** The Frequency of the Patient’s Oral Mucosa Examination and Correct Knowledge Regarding Oral Cancer

Variables		N	%
Which of the following is considered an early symptom /sign of oral cancer?	Right	69	67%
	Wrong	34	33%
What would you consider the most risky site for oral cancer?	Right	77	75%
	Wrong	26	25%
What do you consider the most important risk factor for oral cancer?	Right	56	54%
	Wrong	47	46%
Which of the following has high malignant potential?	Right	42	41%
	Wrong	61	59%
What is the major factor affecting the prognosis of oral cancer?	Right	99	96%
	Wrong	4	4%
What would you do to a patient with an unhealed ulcer persisting for more than 2 weeks?	Right	60	58%
	Wrong	43	42%
What is the treatment plan for leukoplakia?	Right	68	66%
	Wrong	35	34%
General knowledge	Right	69	67%
	Wrong	34	33%

We found no significant associations between the age groups and the correct responses to the knowledge questions (Table 3).

**Table 3:** Associations Between the Age Groups and the Correct Knowledge About Oral Cancer

Variables		20-22 year		23-25 year		Above 25 year		p-value
		N	%	N	%	N	%	
How often do you examine patient's oral mucosa?	Right	18	82%	63	78%	1	50%	0.276
	Wrong	2	9%	18	22%	1	50%	
Which of the following is considered an early symptom /sign of oral cancer?	Right	9	41%	59	73%	1	50%	0.05
	Wrong	11	50%	22	27%	1	50%	
What would you consider the riskiest site for oral cancer?	Right	16	73%	60	74%	1	50%	0.618
	Wrong	4	18%	21	26%	1	50%	
What do you consider the most important risk factor for oral cancer?	Right	12	55%	43	53%	1	50%	0.850
	Wrong	8	36%	38	47%	1	50%	
Which of the following has high malignant potential?	Right	11	50%	30	37%	1	50%	0.330
	Wrong	9	41%	51	63%	1	50%	
What is the major factor affecting the prognosis of oral cancer?	Right	18	82%	79	98%	2	100%	0.284
	Wrong	2	9%	2	2%	0	0%	
What would you do to a patient with an unhealed ulcer persisting for more than 2 weeks?	Right	12	55%	47	58%	1	50%	0.959
	Wrong	8	36%	34	42%	1	50%	
What is the treatment plan for leukoplakia?	Right	14	64%	53	65%	1	50%	0.826
	Wrong	6	27%	28	35%	1	50%	
General knowledge	Right	14	63%	54	67%	1	56%	0.190
	wrong	6	28%	27	33%	1	44%	

Alternatively, there were statistically significant associations between the correct responses and the year of the study regarding early symptoms/signs of oral cancer (p=0.0001), lesions with high malignant potential (p=0.0001), and unhealed ulcer management (p=0.015). Compared to the fourth- and fifth-year students, the dental interns showed more knowledge about early symptoms/signs of oral cancer, while they had less knowledge about the lesions with a high malignant potential (15%), and the management of a patient with unhealed ulcers persisting more than two weeks (38%) (Table 4).

**Table 4:** Associations Between the Study Year and the Correct Knowledge About Oral Cancer * Significant at p value < 0.05

Variables		Fourth-year dental student		Fifth-year dental student		Dental intern		p-value
		N	%	N	%	N	%	
How often do you examine patient's oral mucosa?	Right	32	80%	24	83%	26	76%	0.824
	Wrong	8	20%	5	17%	8	24%	
Which of the following is considered an early symptom/sign of oral cancer?	Right	19	48%	19	66%	31	91%	0.0001*
	Wrong	21	53%	10	34%	3	9%	
What would you consider the most risky site for oral cancer?	Right	29	73%	21	72%	27	79%	0.747
	Wrong	11	28%	8	28%	7	21%	
What do you consider the most important risk factor for oral cancer?	Right	25	63%	17	59%	14	41%	0.160
	Wrong	15	38%	12	41%	20	59%	
Which of the following has high malignant potential?	Right	19	48%	18	62%	5	15%	0.0001*
	Wrong	21	53%	11	38%	29	85%	
What is the major factor affecting the prognosis of oral cancer?	Right	38	95%	28	97%	33	97%	0.892
	Wrong	2	5%	1	3%	1	3%	
What would you do to a patient with an unhealed ulcer persisting for more than 2 weeks?	Right	27	68%	20	69%	13	38%	0.015*
	Wrong	13	33%	9	31%	21	62%	
What is the treatment plan for leukoplakia?	Right	23	58%	19	66%	26	76%	0.228
	Wrong	17	43%	10	34%	8	24%	
General knowledge	Right	27	66%	21	72%	22	64%	0.962
	Wrong	14	34%	8	28%	12	36%	

## Discussion

Oral cancer is a very common malignancy in Saudi Arabia and is emerging as a major health problem. Unfortunately, the majority of oral cancer cases are diagnosed in advanced stages with poor outcomes [3].

Early diagnosis of oral cancer is the key factor in successful treatment and reducing the associated mortality and morbidity rates. A comprehensive understanding and identification of the early changes in oral mucosa and the potentially malignant lesions that precede cancer help reduce the frequency of malignant lesions [11]. Fortunately, most of the participants (96%) in our study recognized the well-known fact that early diagnosis is the most important factor affecting the prognosis of oral cancer.

Good levels of knowledge and attitudes toward early diagnosis of cancer have an important impact on the practices of the dental practitioners [8]. Dentists are among the first professionals who have a chance to screen the oral mucosa. Familiarity with the early signs of cancer helps them to identify, refer, or treat patients in the early stages of oral cancer [12].

The current survey revealed a moderate level (67%) of general knowledge about different aspects of oral cancer among dental students and interns at Dentistry College, PNU, Saudi Arabia. A comparable study at Jazan University, Saudi Arabia revealed a similar moderate level of knowledge among final-year dental students, interns, and faculty members [13]. However, another study at Jazan University detected a poor level of knowledge among oral health practitioners [8]. Alsaud et al. [14] also reported a poor level of knowledge among dental undergraduates and practitioners in Jeddah, Saudi Arabia. In Kuwait, Nazar et al. have reported different levels of knowledge among primary oral health care dentists [15] and newly graduated dentists [16]. Excellent level of general knowledge (81.9%) regarding various aspects of oral cancer has been reported among third- to fifth-year undergraduate dental students in India [17].

In this study, about two-thirds (67%) of the participants correctly identified the presence of an area of erythema in suspicious sites or an unhealed ulcer for more than two weeks as early signs of oral cancer. However, awareness regarding erythroplakia as lesions with high malignant potential was found to be alarmingly low (41%). This knowledge gap necessitates educational interventions in various undergraduate subjects like oral pathology, oral medicine, and oral surgery. Some teaching methods such as problem-based learning would enable students to enhance their knowledge, ascertain the problems, and develop effective skills for resolving these problems [18]. Furthermore, continuous dental education courses are necessary to improve the knowledge of students. In addition, the interns can be involved in educational and training sessions, with case discussions at the hospital. Adequate training encourages conducting a comprehensive oral examination for early and effective diagnosis [19]. Continued undergraduate dental education in oral cancer and well-trained trained oral health providers can help in the early detection of oral cancer, thereby reducing rates of morbidity and increasing oral cancer survival rates [20].

The development of effective prevention strategies for oral cancer dictates a thorough knowledge of all the risk factors. In this study, more than half (54%) recognized that smoking, smokeless tobacco, and mixed white or red lesions in multiple sites are important risk factors for oral cancer. In a comparable study in Nepal, dental students and dentists recognized smoking and chewing tobacco as risk factors for oral cancer [21]. A corresponding study at Princess Nourah bint Abdulrahman University revealed that most healthcare professionals also identified that tobacco use is one of the main risk factors, particularly when combined with alcohol [19]. Halawany et al. [22] reported that more than 95% of dental students identified the association between tobacco smoking and oral cancer in their survey study which included Saudi Arabia, the United Arab Emirates, Yemen, and India Asian countries. It has been reported that smoked and smokeless tobacco products contain carcinogenic compounds that have been associated with mutations in several genes, decreased apoptosis, and increased angiogenesis. Other negative effects of smoking on immunity and dietary habits can increase the risk of cancer [23]. In addition, the use of smokeless tobacco, known as Shammah and Khat, increases the risk of oral cancer among the Saudi population because of prolonged exposure and absorption of these carcinogenic chemicals by oral mucosa [24].

In the Dental Colleges, there is an increased clinical exposure of students to the clinical cases as the clinical year progresses. Thus, dental interns are expected to have a higher level of awareness and practical skills [25]. Our study revealed contradictory results; the interns, compared to the fourth- and fifth-year students, showed significantly higher knowledge about early symptoms and signs of oral cancer, while they had less knowledge about the lesions with high malignant potential and the management plan of unhealed ulcers persisting more than two weeks. Consistent with the present finding, a previous study in Iran found that only half of the participating dentists were aware of the precancerous lesions [26]. Another study in Jordan detected lower levels of knowledge regarding the oral potentially malignant lesions among all dental students [27].

The detected lack of knowledge about precancerous lesions and their management plan is an alarming finding that should be urgently addressed to minimize the incidence of oral cancer. It is the responsibility of dental colleges to ensure a basic knowledge of the students about the risk factors, clinical picture, suspicious mucosal changes, prevention, and treatment plans. However, the highlighted knowledge gap among the interns in this study requires continuous post-academic education and training workshops to improve their future diagnostic ability and early detection practices. Furthermore, Shamala et al. [28] have recently suggested exposure of undergraduate students to the evolving artificial intelligence technologies that showed favorable results in oral cancer screening and detection.

The study has some limitations, such as the cross-sectional design, where a cause-effect relationship could not be confirmed, and the non-probability sampling technique that significantly limits the extrapolation of the results. Additionally, the use of an online, anonymously filled-in questionnaire possibly causes errors in data collection, with over or under-reporting of results.

## Conclusions

The findings of the present study indicate that the level of knowledge of undergraduate dental students and dental interns is moderate (67%) and not satisfactory. The participants were aware of the importance of early diagnosis for better outcomes, early diagnostic signs, and risk factors. A high proportion (96%) of the participants realized that early diagnosis has a good impact on the prognosis of oral cancer. However, there was insufficient awareness of the potentially malignant lesions and the treatment plans for unhealed chronic ulcers, particularly among the interns. Less than half (41%) correctly identified the lesions with high malignant potential and only 58% of participants were knowledged about the management of persistent ulcers. Knowledge about the risk factors was also insufficient where 54% of participants recognized that smoking, smokeless tobacco, and mixed white or red lesions in multiple sites are important risk factors for oral cancer. The highlighted significant gaps in the awareness of some aspects of oral cancer need to be reinforced through the development of undergraduate dental curricula and continuing education programs and workshops to be able to adequately diagnose early oral cancerous lesions during routine screening.
